# Gaps in Guideline-Concordant Care in Diabetic Kidney Disease: A Real-World Analysis of Screening, Treatment, and Associated Factors

**DOI:** 10.3390/healthcare14142237

**Published:** 2026-07-22

**Authors:** Abdullah H. Almalki, Majed Alharthi, Ahmad Makeen, Mohamed E. Balla, Ashraf Marwan, Eman Kotbi, Sarah Dahlan, Turki Banamah, Muhammad Awais, Reem Baduwaylan, Laila F. Sadagah

**Affiliations:** 1 Nephrology Section, Department of Medicine, King Abdulaziz Medical City, Ministry of National Guard Health Affairs, P.O. Box 9515, Jeddah 21423, Saudi Arabia; 2 College of Medicine, King Saud Bin Abdulaziz University for Health Sciences, Ministry of National Guard Health Affairs, P.O. Box 9515, Jeddah 21423, Saudi Arabia; 3 King Abdullah International Medical Research Center (KAIMRC), Ministry of National Guard Health Affairs, P.O. Box 9515, Jeddah 21423, Saudi Arabia

**Keywords:** diabetic kidney disease, chronic kidney disease, guideline-concordant care, albuminuria screening, RAAS inhibitors, quality of care, care cascade, Saudi Arabia

## Abstract

**Highlights:**

**What are the main findings?**

- Only 22.7% of adults with diabetes received full guideline-concordant DKD care; the gap was more evident in screening than in treatment, with RAASi prescribed in 78.9% of patients once albuminuria was identified.
- Primary care management was the strongest independent predictor of full pathway completion (OR 4.79, 95% CI 3.45–6.66; *p* < 0.001); patient demographics and kidney function were not.

**What are the implications of the main findings?**

- Care delivery tracked setting rather than patient characteristics, indicating system-level barriers open to structural intervention.
- Embedding ACR and HbA1c ordering into default clinical workflows is the most actionable improvement target.

**Abstract:**

**Background:** Diabetic kidney disease (DKD) is the leading cause of chronic kidney disease and end-stage renal disease worldwide. Despite robust evidence-based guidelines, real-world adherence to recommended preventive care remains inadequate. Most prior evaluations have focused on individual care components rather than comprehensive composite care delivery. **Methods:** A retrospective cross-sectional study of 1010 adult patients with diabetes attending outpatient clinics at King Abdulaziz Medical City, Ministry of National Guard Health Affairs (MNGHA), Jeddah, Saudi Arabia. Full guideline-concordant care (GCC) was defined as composite completion of HbA1c monitoring, albuminuria screening using the urine albumin-to-creatinine ratio (ACR), and renin–angiotensin–aldosterone system inhibitor (RAASi) use among patients with detected albuminuria. Multivariable logistic regression (*n* = 1007) identified independent predictors of full GCC. **Results:** Only 22.7% (95% CI: 20.1–25.3%) of patients achieved full GCC. Partial care was the most common pattern (46.2%), followed by no care (20.7%) and near-complete care (10.4%). HbA1c testing was performed in 55.0% and albuminuria screening in 49.3% of patients. Among those with detected albuminuria, RAASi therapy was prescribed in 78.9%. Primary care management (OR 4.79, 95% CI 3.45–6.66; *p* < 0.001) and hypertension (OR 2.00, 95% CI 1.33–3.00; *p* = 0.001) were independently associated with full GCC. Age, gender, kidney function, and cardiac disease were not significant predictors. **Conclusions:** Substantial gaps exist in guideline-recommended DKD care, driven primarily by screening deficiencies rather than treatment failures. Care delivery is influenced more by healthcare setting than by patient characteristics, highlighting the need for system-level interventions targeting detection infrastructure across all care settings.

## 1. Introduction

Diabetic kidney disease (DKD) is the most common cause of chronic kidney disease (CKD) globally and accounts for approximately 40% of all cases of end-stage renal disease (ESRD) [1]. It is a serious microvascular complication of both type 1 and type 2 diabetes mellitus, characterised by progressive albuminuria and declining glomerular filtration rate, ultimately leading to kidney failure, cardiovascular morbidity, and premature mortality [2]. The global burden of DKD has expanded in parallel with the worldwide diabetes epidemic, with significant implications for healthcare systems, particularly in regions with high diabetes prevalence such as the Arabian Gulf [3].

Saudi Arabia faces one of the highest burdens of type 2 diabetes globally, with a national prevalence exceeding 18% in adults [4]. This creates an urgent need for effective early detection and management of DKD in order to slow disease progression and reduce the downstream burden on renal replacement therapy services. International clinical practice guidelines from the Kidney Disease: Improving Global Outcomes (KDIGO) organisation and the American Diabetes Association (ADA) provide clear, evidence-based recommendations for the management of DKD. These include annual monitoring of glycated haemoglobin (HbA1c), routine screening for albuminuria using the urine albumin-to-creatinine ratio (ACR), and initiation of renin–angiotensin–aldosterone system (RAAS) inhibitors in patients with detected albuminuria to reduce the rate of kidney function decline and cardiovascular risk [5,6].

Despite the strength of these recommendations and their endorsement across major guidelines, real-world adherence to guideline-recommended care in DKD remains consistently suboptimal. Studies from diverse healthcare settings have documented significant gaps in albuminuria screening rates, HbA1c monitoring, and appropriate pharmacological management [7,8]. These deficiencies translate into missed opportunities for early intervention and potentially avoidable progression to advanced kidney disease. However, most prior studies have examined individual care components in isolation rather than evaluating the delivery of comprehensive, composite care, limiting the ability to identify patients who are receiving fully adequate management.

Furthermore, the factors associated with guideline adherence in DKD are incompletely understood, particularly in non-Western healthcare contexts. Patient-level factors such as age, sex, and comorbidities, as well as system-level factors including care setting and provider type, may differentially influence whether patients receive recommended care. Understanding these drivers is essential for designing targeted quality improvement interventions [9].

The present study addresses these gaps by evaluating the prevalence of composite guideline-concordant care (GCC)—encompassing HbA1c monitoring, albuminuria screening, and RAASi use—in a large cohort of patients with diabetes attending a tertiary healthcare centre in Jeddah, Saudi Arabia. We also sought to identify patient- and system-level predictors of full GCC delivery using multivariable logistic regression, with the aim of informing locally relevant quality improvement strategies. This study represents a secondary analysis of a dataset from a previously published observational study conducted at the same institution [10].

## 2. Methods

### 2.1. Study Design and Setting

This retrospective cross-sectional study was conducted at King Abdulaziz Medical City, Ministry of National Guard Health Affairs (MNGHA), Jeddah, Saudi Arabia. MNGHA is a comprehensive tertiary healthcare system delivering care across primary, secondary, and tertiary levels. Data were derived from a secondary analysis of an Institutional Review Board–approved dataset (Study No. SP16/208/J), approved by the King Abdullah International Medical Research Center (KAIMRC). The KAIMRC Institutional Review Board reviewed and granted ethical approval for the study (Study No. SP16/208/J). Informed consent was waived due to the retrospective and fully anonymised nature of the data. Data for the study were assembled over two collection phases. An initial sample accrued through 2018 was limited in size and was subsequently augmented by a second phase of 568 additional patients collected after the COVID-19 pandemic, once pandemic-related disruption to routine outpatient attendance had resolved. The full analytic cohort therefore spans two calendar periods, 2016–2018 and 2021–2023. The present analysis pursued a distinct research objective focused on composite GCC and its associated factors, which were not addressed in the original published study [10]. Specifically, the present analysis draws on the same institutional cohort and the same underlying care-process variables (HbA1c testing, ACR screening, and RAASi prescription) as the earlier report; however, whereas that report described the rate of each process individually, the current study newly derives a composite full-GCC outcome and models its patient- and system-level correlates, analyses that do not overlap with the prior publication.

### 2.2. Study Population

Adult patients (≥18 years) with an established diagnosis of diabetes mellitus who were attending outpatient care in primary healthcare, general internal medicine, or endocrinology clinics within MNGHA were eligible for inclusion. Patients were required to have documented clinical follow-up and available laboratory data. Exclusion criteria included: established CKD under active nephrology care, advanced kidney dysfunction (eGFR < 30 mL/min/1.73 m^2^), patients on renal replacement therapy, kidney transplant recipients, and pregnant patients. These criteria were designed to focus the analysis on patients in the early-to-moderate stages of DKD where preventive and monitoring interventions are most relevant. The study was thus designed to evaluate early DKD detection and preventive management across the broader diabetes population—including patients without established nephropathy—rather than the care of advanced or overt DKD; patients already receiving nephrology care were deliberately excluded. The relatively preserved mean kidney function of the cohort reflects this intended focus on screening and early intervention rather than any limitation of case ascertainment.

### 2.3. Data Collection

Data were extracted retrospectively from electronic medical records using a standardised collection form developed for the original study protocol. Variables included: demographic characteristics (age, gender), clinical comorbidities (hypertension, cardiac disease), care setting (primary versus tertiary care), laboratory values (HbA1c, eGFR, urine ACR), and process-of-care documentation (HbA1c testing, albuminuria screening, RAASi prescription).

### 2.4. Definition of Guideline-Concordant Care

A composite measure of guideline-concordant care (GCC) was constructed based on established guideline recommendations for DKD management [5,6]. The composite included three components: (1) HbA1c monitoring—documentation of at least one HbA1c measurement during the follow-up period; (2) albuminuria screening—documentation of urine ACR measurement; and (3) RAASi use—prescription of an angiotensin-converting enzyme inhibitor (ACEi) or angiotensin receptor blocker (ARB) among patients with albuminuria detected on available ACR testing (ACR ≥ 3 mg/mmol, equivalent to ≥30 mg/g). Full GCC was defined as achievement of all applicable components, yielding a binary outcome (full care vs. not full care) for analysis. Applicability was assigned per patient: HbA1c monitoring and ACR screening applied to all patients, whereas the RAASi component applied only to patients in whom albuminuria was detected. Patients without detected albuminuria were therefore eligible for full GCC on the basis of completed HbA1c monitoring and ACR screening alone, whereas patients who were not screened for albuminuria could not meet the composite. Consistent with this scheme, the 229 patients classified as receiving full GCC comprised 149 patients without detected albuminuria (HbA1c and ACR both completed) and 80 patients with detected albuminuria who additionally received RAASi therapy. RAASi therapy was selected as the pharmacological indicator of treatment because it has been a cornerstone guideline recommendation for over two decades, allowing consistent evaluation across the study period. Newer agents, including SGLT2 inhibitors and non-steroidal mineralocorticoid receptor antagonists, were not incorporated into the composite because their guideline adoption occurred unevenly across the two collection periods, whereas RAASi therapy remained a stable and universally recommended cornerstone of albuminuric DKD management throughout; restricting the pharmacological component to RAASi therefore allowed a single, consistent treatment criterion to be applied uniformly across the entire study span.

### 2.5. Statistical Analysis

Statistical analysis was performed using IBM SPSS Statistics (version 26, IBM Corp., Armonk, NY, USA). Continuous variables were expressed as mean ± standard deviation (SD) and compared between groups using independent-samples *t*-tests. Categorical variables were summarised as frequencies and percentages and compared using chi-square tests, with Fisher’s exact test applied where appropriate for 2 × 2 tables. A multivariable logistic regression model was constructed to identify factors independently associated with full GCC. Covariates entered into the model included: age (per 10-year increment), gender (female vs. male), care level (primary vs. tertiary), hypertension (yes vs. no), cardiac disease (yes vs. no), and eGFR (per 10 mL/min/1.73 m^2^ increment). Results were expressed as odds ratios (OR) with 95% confidence intervals (CI). Three cases with missing covariate data were excluded from the regression model, leaving 1007 patients in the multivariable analysis. Model calibration was assessed with the Hosmer–Lemeshow goodness-of-fit test, and explanatory power with Nagelkerke R^2^. A two-sided *p*-value < 0.05 was considered statistically significant.

## 3. Results

### 3.1. Baseline Characteristics

A total of 1010 patients were included. The mean age was 61.9 ± 12.0 years, and 57.6% were female. Hypertension was present in 72.8% and cardiac disease in 14.2% of the cohort. Primary care was the managing setting for 30.0% of patients. The mean eGFR was 88.0 ± 18.2 mL/min/1.73 m^2^ and the mean HbA1c was 7.92 ± 1.70%. Baseline characteristics stratified by GCC status are presented in Table 1.

Patients who achieved full GCC did not significantly differ from those who did not in terms of age (62.4 ± 10.8 vs. 61.8 ± 12.4 years; *p* = 0.517), eGFR (86.5 ± 18.1 vs. 88.4 ± 18.3 mL/min/1.73 m^2^; *p* = 0.157), or ACR (*p* = 0.789). Female patients were less likely to achieve full GCC compared to males (119/582, 20.4% vs. 110/428, 25.7%; Pearson chi-square *p* = 0.049; Fisher’s exact *p* = 0.057), a difference of borderline statistical significance. Patients managed in primary care were significantly more likely to receive full GCC (126/303, 41.6%) than those managed in tertiary care (103/707, 14.6%; *p* < 0.001). Hypertension was more prevalent among those achieving full GCC (78.2% vs. 71.2%; *p* = 0.037). Patients with full GCC had significantly lower mean HbA1c (7.67 ± 1.51% vs. 8.09 ± 1.80%; *p* = 0.004).

### 3.2. Distribution of Guideline-Concordant Care

Overall, only 22.7% (95% CI: 20.1–25.3%) of patients achieved full GCC across all applicable care components. The majority (77.3%, 95% CI: 74.7–79.9%) had at least one missed component. Partial care was the most common pattern, observed in 46.2% (95% CI: 43.1–49.3%) of patients. A further 20.7% (95% CI: 18.2–23.2%) received none of the evaluated care processes, while 10.4% (95% CI: 8.5–12.3%) achieved near-complete care with one missed component.

### 3.3. Adherence to Individual Components of Care

HbA1c monitoring was documented in 55.0% (95% CI: 51.9–58.1%) of the overall cohort, while albuminuria screening with ACR was performed in 49.3% (95% CI: 46.2–52.4%). Among the 498 patients with available albuminuria data, 37.1% (95% CI: 32.8–41.4%) had detected albuminuria. Of those with detected albuminuria (*n* = 185), 78.9% (95% CI: 73.0–84.8%) were receiving RAASi therapy. These figures are summarised in Table 2. These data demonstrate that deficiencies in guideline adherence are concentrated at the screening level, whereas treatment uptake among those already identified as high-risk is comparatively robust.

### 3.4. Factors Associated with Full Guideline-Concordant Care

**Figure 1:**
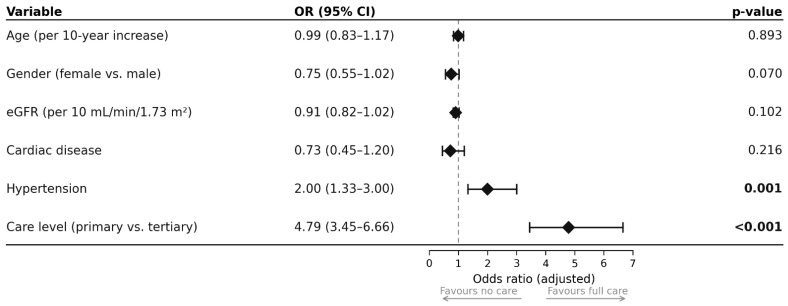
Factors associated with full guideline-concordant care (forest plot of adjusted odds ratios). Forest plot of adjusted odds ratios (OR) with 95% confidence intervals (CI) from multivariable logistic regression. The dependent variable was full guideline-concordant care. Tertiary care was the reference category for care level. Age and eGFR were modelled per 10-unit increase. The arrow for care level indicates that the upper confidence limit (6.66) extends beyond the axis. OR: odds ratio; CI: confidence interval; eGFR: estimated glomerular filtration rate.

Results of the multivariable logistic regression analysis, conducted among 1007 patients with complete covariate data, are illustrated in Figure 1. Age (per 10-year increase: OR 0.99, 95% CI 0.83–1.17; *p* = 0.893), gender (OR 0.75, 95% CI 0.55–1.02; *p* = 0.070), cardiac disease (OR 0.73, 95% CI 0.45–1.20; *p* = 0.216), and eGFR (OR 0.91, 95% CI 0.82–1.02 per 10 mL/min/1.73 m^2^; *p* = 0.102) were not independently associated with achieving full GCC.

In contrast, care level and hypertension were significant independent predictors. Patients managed in primary care had nearly five-fold higher odds of receiving full GCC compared with those in tertiary care (OR 4.79, 95% CI 3.45–6.66; *p* < 0.001). Hypertension was also independently associated with full GCC (OR 2.00, 95% CI 1.33–3.00; *p* = 0.001). The model showed acceptable calibration (Hosmer–Lemeshow *p* = 0.588) with modest explanatory power (Nagelkerke R^2^ = 0.15).

## 4. Discussion

This real-world study demonstrates that comprehensive, guideline-concordant care for DKD is achieved in fewer than one-quarter of patients with diabetes managed at a large tertiary healthcare system in Saudi Arabia. This finding underscores a substantial quality gap in the delivery of care to a high-risk population, and extends prior work by quantifying adherence using a composite measure that simultaneously evaluates monitoring, screening, and treatment. Whereas the original study at this institution reported individual care component rates in isolation [10], the present analysis reframes the question around integrated pathway completion. This reframing reveals a clinically actionable finding: treatment uptake is relatively preserved once patients are identified (RAASi in 78.9% of albuminuric patients), shifting the primary intervention target from prescribing behaviour to detection infrastructure. The multivariable analysis of composite GCC and its system-level correlates was not examined in the prior publication.

The low rates of HbA1c monitoring (55.0%) and albuminuria screening (49.3%) observed in this study are consistent with findings from other healthcare settings. A large multicentre analysis similarly reported that fewer than half of patients with type 2 diabetes completed guideline-recommended albuminuria testing in primary care [7]. Similarly, a large national general-practice study reported that fewer than half of adults with diabetes had their HbA1c assessed over a 12-month period [11]. The identification that screening gaps—rather than treatment deficiencies—are the primary driver of incomplete GCC in our cohort is clinically significant. Among patients who were screened and found to have albuminuria, RAASi therapy was prescribed in 78.9%, indicating that when high-risk patients are identified, treatment is relatively well implemented. This suggests that, although both accurate detection and appropriate treatment are essential to the management of diabetes and DKD, the greater opportunity for improvement in this cohort lies at the detection (screening) stage rather than in prescribing behaviour. This inference applies specifically to patients who were screened and identified as albuminuric; because more than half of the cohort did not undergo ACR testing, some unscreened patients may have had undetected albuminuria and correspondingly missed treatment, so the apparent preservation of treatment uptake should be read as conditional on detection rather than as evidence of uniformly adequate prescribing.

The most striking predictor of full GCC in our cohort was care setting. Patients managed in primary care had nearly five-fold higher odds of achieving full GCC compared with those in tertiary care clinics. This finding is counterintuitive given the perception that specialist care provides more comprehensive management. However, we hypothesise that it reflects the structure of primary care at MNGHA, where standardised chronic disease management protocols—including periodic HbA1c and ACR monitoring—are embedded into routine follow-up workflows for patients with diabetes. Because workflow-level variables were not captured in this dataset, this explanation is advanced as a hypothesis rather than a demonstrated mechanism [12]. Tertiary clinics, by contrast, may focus on specific clinical problems at the time of referral without systematically ensuring completion of all guideline-recommended screening components. This observation aligns with evidence from other settings demonstrating that structured primary care approaches outperform specialist-based episodic care for the delivery of preventive services in chronic disease [13].

The association between hypertension and full GCC is also noteworthy. Patients with hypertension had double the odds of receiving complete guideline-directed care. This likely reflects greater clinical engagement and more frequent monitoring in patients with multiple comorbidities who are already established on antihypertensive medications—many of which are RAASi—and who require more structured follow-up. The co-management of hypertension in patients with diabetes may serve as a proxy for more systematic disease management, which also encompasses DKD-related care processes [14].

In contrast to care setting and hypertension, patient-level factors including age, gender, eGFR, and cardiac disease were not independently associated with GCC. This finding suggests that the observed gaps in care reflect systemic or structural barriers within the healthcare delivery environment rather than differential provider behaviour based on patient characteristics. From an equity standpoint, this is somewhat reassuring, indicating that patients are not systematically receiving lower-quality care based on demographics. However, the borderline association between female gender and lower rates of full GCC (Pearson *p* = 0.049; Fisher’s exact *p* = 0.057 in univariable analysis; OR 0.75, *p* = 0.070 in multivariable analysis) warrants further investigation in larger studies.

The overall low Nagelkerke R^2^ of 0.15 indicates that the variables included in our model explain only a modest proportion of the variability in GCC delivery. This suggests that other unmeasured factors—such as physician knowledge and awareness, clinical workflow design, availability of clinical decision support tools, patient health literacy and engagement, and organisational priorities—likely play important roles [15]. Future studies should incorporate these factors to develop more comprehensive explanatory models of care quality in DKD.

The implications of these findings for quality improvement are clear. Given that care setting is the dominant modifiable predictor of GCC, system-level interventions are likely to yield the greatest impact. Implementation strategies such as electronic health record (EHR)-embedded clinical decision support tools that prompt ordering of HbA1c and ACR tests, registry-based chronic disease monitoring programmes, and performance dashboards with feedback to clinicians have demonstrated efficacy in improving guideline adherence in DKD and related conditions [16,17]. Extending structured primary care protocols to tertiary clinic environments—or implementing integrated care models—may help reduce the disparity in care delivery observed between settings in this study.

### Strengths and Limitations

This study has several strengths, including a large well-characterised cohort, the use of a composite GCC measure that captures the full care pathway, and multivariable analysis to adjust for potential confounders. The real-world, single-centre design provides relevant insights into care delivery at a major healthcare system serving a predominantly Saudi population. However, several limitations should be acknowledged. As a cross-sectional analysis based on retrospective chart review, causal inferences cannot be drawn. The study was conducted at a single tertiary centre, which may limit generalisability. It is possible that some screening or monitoring was performed at external facilities and not captured in the electronic records, potentially leading to underestimation of true adherence rates. The dataset did not include information on patient-reported factors such as health literacy, clinic attendance, or socioeconomic status, which may confound care delivery. Other potentially important confounders—including diabetes duration and type, number of clinic visits, reason for tertiary referral, disease complexity, and contraindications to or intolerance of RAASi therapy—were likewise not captured, so residual confounding of the care-setting association cannot be excluded. The treatment component was restricted to RAASi therapy, as the adoption of SGLT2 inhibitors and non-steroidal MRAs occurred unevenly across the two collection periods; future studies should incorporate these newer agents. Finally, the original dataset was collected under a protocol approved in 2016 and care practices may have since evolved with updated guidelines and quality improvement initiatives. In addition, because the cohort was assembled across two collection periods, documentation completeness differed between phases, with less complete process documentation in the earlier phase; this may have led to underestimation of care delivery in that subset and should be borne in mind when interpreting the pooled estimates. Finally, albuminuria was based on available ACR values without a mandated repeat measurement, so transient causes of albuminuria cannot be fully excluded.

## 5. Conclusions

This study identifies substantial and clinically meaningful gaps in the delivery of comprehensive guideline-concordant care for DKD in a tertiary healthcare setting in Saudi Arabia. Only 22.7% of patients achieved full guideline-directed care, with deficiencies concentrated at the screening level rather than in pharmacological treatment. Primary care management and hypertension were the dominant independent predictors of full GCC, while patient demographics and kidney function were not significant drivers. These findings highlight the strong association between care setting and the quality of DKD management; because the analysis is cross-sectional, these associations should not be interpreted as demonstrating that system design causally determines care quality. System-level interventions—particularly those that embed standardised monitoring protocols into clinical workflows across all care settings—represent the most promising strategy for improving GCC delivery, reducing avoidable DKD progression, and alleviating the long-term burden of end-stage renal disease in this population.

## Figures and Tables

**Table 1:** Baseline characteristics by guideline-concordant care status.

Variable	Overall (*n* = 1010)	Full GCC (*n* = 229)	Not Full GCC (*n* = 781)	*p*-Value
Age, years, mean ± SD	61.9 ± 12.0	62.4 ± 10.8	61.8 ± 12.4	0.517
Female gender, *n* (%)	582 (57.6)	119 (52.0)	463 (59.3)	0.049
Primary care setting, *n* (%)	303 (30.0)	126 (55.0)	177 (22.7)	<0.001
Hypertension, *n* (%)	735 (72.8)	179 (78.2)	556 (71.2)	0.037
Cardiac disease, *n* (%)	143 (14.2)	27 (11.8)	116 (14.9)	0.242
eGFR, mL/min/1.73 m^2^, mean ± SD	88.0 ± 18.2	86.5 ± 18.1	88.4 ± 18.3	0.157
ACR, mg/mmol, mean ± SD *	7.57 ± 18.64	7.81 ± 17.60	7.36 ± 19.51	0.789
HbA1c, %, mean ± SD *	7.92 ± 1.70	7.67 ± 1.51	8.09 ± 1.80	0.004

Data are presented as mean ± SD or number (%). Comparisons by independent samples *t*-test or chi-square test as appropriate. * HbA1c and ACR values reported among patients with available measurements only. SD: standard deviation; eGFR: estimated glomerular filtration rate; ACR: albumin-to-creatinine ratio; HbA1c: glycated haemoglobin; GCC: guideline-concordant care.

**Table 2:** Adherence to individual components of guideline-concordant care (*n* = 1010).

Care Process Component	*n/N*	Proportion, % (95% CI)
HbA1c monitoring	555/1010	55.0 (51.9–58.1)
Albuminuria (ACR) screening	498/1010	49.3 (46.2–52.4)
Albuminuria detected (ACR ≥ 3 mg/mmol) *	185/498	37.1 (32.8–41.4)
RAASi use among albuminuric patients †	146/185	78.9 (73.0–84.8)
Full GCC achieved	229/1010	22.7 (20.1–25.3)

* Calculated among patients with available ACR testing (*n* = 498). † Calculated among patients with detected albuminuria (*n* = 185). RAASi: renin–angiotensin–aldosterone system inhibitors (ACE inhibitors or angiotensin receptor blockers); ACR: albumin-to-creatinine ratio; GCC: guideline-concordant care; CI: confidence interval.

## Data Availability

The data presented in this study are available on request from the corresponding author. The data are not publicly available due to privacy and ethical restrictions, as they comprise de-identified patient-level clinical records governed by the institutional data governance regulations of KAIMRC and the Ministry of National Guard Health Affairs, under which the study was approved (IRB SP16/208/J).
